# Study of meat quality and flavour in different cuts of Duroc‐Bamei binary hybrid pigs

**DOI:** 10.1002/vms3.409

**Published:** 2020-12-16

**Authors:** Guoshun Chen, Yu Cai, Yingyu Su, Dong Wang, Xiaolong Pan, Xijun Zhi

**Affiliations:** ^1^ College of Animal Science and Technology Gansu Agricultural University Lanzhou China; ^2^ College of Animal Science and Technology Nanjing Agricultural University Nanjing China; ^3^ Gansu Heisiling Agriculture and Animal Husbandry Technology Co., Ltd. Dingxi China; ^4^ Qingshui Jinsang Agriculture and Animal Husbandry Technology Co., Ltd. Tianshui China

**Keywords:** Du‐Ba binary hybrid pig, flavour substances, meat quality

## Abstract

**Background:**

Meat quality and flavour are important criteria for judging fresh pork and processed products. However, there have been few studies on meat quality and volatile flavour substances of different parts of binary hybrid pigs.

**Objective:**

To study the differences in meat quality and volatile flavour substances between different cuts of pork, which could provide the basis for consumer decision‐making when purchasing pork.

**Methods:**

Twenty Du‐Ba binary hybrid pigs (first filial [F1] generation) bred from Duroc and Bamei pigs were used. This study systematically compared and analysed the basic nutritional components, amino acid composition, fatty acid composition and flavour profiles of *longissimus dorsi*, rib muscle and tendon meat of four Du‐Ba binary hybrid pigs; all assays were repeated in triplicate.

**Results:**

Crude protein, calcium and phosphorus content in tendon meat were higher than that in the *longissimus dorsi*. The intramuscular fat content of the rib muscle was higher than that in the *longissimus dorsi* and tendon meat (*p* < 0.05). The amino acid content was highest in the tendon meat. The levels of essential amino acids and flavour‐associated amino acids per kilogram of *longissimus dorsi* were higher than those in the rib muscle and tendon meat. Moreover, the content of aspartic acid, serine and cystine were higher in the *longissimus dorsi* than in the other two parts. The type of saturated fatty acids and the type and content of unsaturated fatty acids in tendon meat were higher than in the *longissimus dorsi* and rib muscle. The total content of volatile flavour compounds was higher in the *longissimus dorsi* than in the rib muscle and tendon meat.

**Conclusion:**

The rib muscle contains high deposits of fat, and tendon meat has a relatively high nutritional value, while the *longissimus dorsi* has a stronger flavour.

## Graphical abstract

The intramuscular fat content of the rib muscle is higher than that of the *longissimus dorsi* and tendon meat. The crude protein content, calcium content, phosphorus content, amino acid content, type of saturated fatty acids, and the type and content of unsaturated fatty acids in tendon meat are higher than those in the *longissimus dorsi* and rib muscle. The UMP content, GMP content, IMP content, flavour‐associated amino acids content, and the total content of volatile flavour compounds in the *longissimus dorsi* are higher than those in the rib muscle and tendon meat.

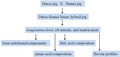

**Highlights**

1. The intramuscular fat content of the rib muscle is higher than that of the *longissimus dorsi* and tendon meat.
2. The crude protein content, calcium content, phosphorus content, amino acid content, type of saturated fatty acids, and the type and content of unsaturated fatty acids in tendon meat are higher than those in the *longissimus dorsi* and rib muscle.
3. The UMP content, GMP content, IMP content, flavour‐associated amino acids content, and the total content of volatile flavour compounds in the *longissimus dorsi* are higher than those in the rib muscle and tendon meat.

## 1 INTRODUCTION

China is the largest pig‐raising country in the world. Pork consumption accounts for more than 60% of the total meat consumption in China (Carvalho‐Salemi et al., 2017). According to the Organization for Economic Development (OECD) and the United Nations Food and Agriculture Organization (FAO) analysis, pork consumption in China will reach 59.3 million tons by 2020 (Hu et al., 2015). The flavour of pork is enhanced by its soft muscle fibres and low levels of connective tissue; these properties have caused it to be favoured by a vast number of consumers (Vlachos et al., 2016). In addition, pork has a high total content of all amino acids (Wolk, 2017). Pork is rich in potassium, iron, magnesium, and other elements (Benet et al., 2015). The B vitamins, especially vitamin B1, are highly abundant in pork (Esteve et al., 2002). Fat is the main nutritional component of pork; this macronutrient is necessary to maintain nutritional balance and enhance the anti‐disease immune function of the human body (Kucha et al., 2018).

Pork quality is primarily determined by the edible and nutritional quality of the meat, which can differ depending on the cut of pork (Alfaia et al., 2019). Pork flavour is mainly composed of water‐soluble and volatile compounds (Wojtasik‐Kalinowska et al., 2016). Meat quality and flavour are important criteria when judging fresh pork and processed products. Both domestic and foreign studies have shown that meat quality indicators differ depending on the cut of pork, and these indicators have become the basis for consumers to select pork (Long et al., 2017; Rosenvold & Andersen, 2003). Xi et al. researched the difference in content of flavour‐providing substances in different muscle parts of Bamei pigs, and the results suggested that compared with pork belly, the *longissimus dorsi* had a lower fat, higher protein, and higher amino acid content, which is more in line with people's demand for high nutritional meat (Xi et al., 2019). Xu et al. tested the meat quality parameters of different carcass parts of ten 100 kg Duroc gilts, and the results indicated that the cut of the pork had a significant effect on some meat quality parameters; compared to the muscles of the ham, the muscles around the shoulder had lower drip loss, muscle fibre diameter, shearing force, and intramuscular fat (Xu et al., 2004).

Studies on pork quality and volatile flavour substances are mostly based on the differences between different breeds or different treatment methods (Lanferdini et al., 2018; Lebret et al., 2018). However, there are few studies on pork quality and volatile flavour substances of different parts of binary hybrid pigs. In this experiment, Du‐Ba binary hybrid pigs were bred from Duroc and Bamei parent pigs using the binary hybrid model (Duroc × Bamei pig). During the feeding period, pigs were fed with high‐quality feed to obtain commercial and ecological high‐quality pork. In order to study the differences in meat quality and volatile flavour substances between different parts of the pig, three different cuts of pork (*longissimus dorsi*, rib muscle, and tendon meat) were selected to provide a reference for the breeding of high‐quality pork breeds and the basis for consumers to purchase pork that meets their demands.

## 2 MATERIALS AND METHODS

### 2.1 Experimental pigs and diets

In this experiment, Du‐Ba binary hybrid pigs (first filial [F1] generation) were bred as the research subjects from Duroc and Bamei pigs with different coat colours. The Du‐Ba binary hybrid pigs were provided by Gansu Black Commander Agriculture and Animal Husbandry Technology Co., Ltd. Twenty Du‐Ba binary hybrid pigs (half male and half female) with the same growth status, health level and body weight (25 ± 2 kg) were randomly selected for the fattening test, and two repeat experiments were performed, with 10 Du‐Ba binary hybrid pigs each. The animal procedures used in this study were reviewed and approved by the Gansu Agricultural University's Academic Committee and the National Natural Science Foundation of China, according to the guidelines established by the Biological Studies Animal Care and Use Committee of Gansu Province (Approval No. 31660670).

During the feeding period, the dietary composition was designed according to the People's Republic of China pig feeding standard (2004) and the nutrient requirements of pigs (NRC, 2012). The ideal protein model was adopted based on standard ileal digestible amino acid balance, and dietary protein level was reduced by 2%. The dietary composition and nutritional levels of the experimental diets are shown in Table 1.

**TABLE 1:** Dietary composition and nutritional levels of experimental diets

Composition ratio of raw materials in CP 14.0% low protein diet		Nutritional components of dietary formula	
Corn	69.00	Digestive energy (≥)	10.52
Soybean meal	14.30	Crude protein (≥)	**16.0% (16.05^b^)**
Wheat bran	4.50	Crude fibre (≤)	**2.68**
Cottonseed meal	1.60	Calcium (≥)	0.62
Bentonite	4.00	Phosphorus (≥)	0.55
Soybean oil	1.70	Feed grade NaCl (≥)	0.37
Enzyme preparation	0.10	SID Lys (≥)	0.75
Lys (98%)	0.32	SID Met (≥)	0.21
Met (98%)	0.24	SID Thr (≥)	0.47
Thr (98%)	0.21	SID Try (≥)	0.13
Try (98%)	0.05	SID Iso (≥)	0.58
Iso (98%)	0.08	SID Leu (≥)	0.77
Leu (98%)	0.07	SID Val (≥)	0.48
Val (98%)	0.09	SID Phe (≥)	0.49
Phe (98%)	0.14		
His (80.1%)	0.07		
Calcium carbonate	1.50		
Calcium hydrogen phosphate	1.18		
0.5% Core material for fattening pigs^a^	0.50		
Feed grade NaCl	0.35		
Total	100.00		

^a^ 1% pig premix nutritional composition was converted into per kilogram compound feed (more than), including Fe (68.0 g), Zn (60.0 mg), Mn (18.0 mg), Cu (4.50 mg), Se (0.3 mg), I (0.14 mg), Vitamin A (3,000 IU), Vitamin D_3_ (380 IU), Vitamin E (52.0 mg), thiamine (2.10 mg), riboflavin (2.40 mg), biotin (0.25 mg), folic acid (0.62 mg), nicotinic acid (30.00 mg), calcium pantothenate (10.50 mg), vitamin B_6_ (1.10 mg), vitamin B_12_ (0.02 mg), choline chloride (400.0 mg) and antioxidant (30.0 mg).

^b^ The energy and amino acid data were calculated theoretically, and others were measured value (in bold).

### 2.2 Slaughtering of pigs and subsequent muscle sampling

Pigs were fattened until 240 days (115 ± 5 kg), at the completion of the feeding trial, all pigs were subjected to overnight fasting with no access to water for 2 hr. To determine meat quality and flavour substances, four pigs with identical growth, with good health and nutrition were randomly selected for slaughter. Within 2 hr after slaughter, *longissimus dorsi* (Tenderloin), rib meat and tendon meat samples were taken from each pig according to the technical specifications of the NY/T821‐2004 pig muscle quality determination, and samples were stored in marked self‐sealed bags and were then vacuum‐packed. Routine nutrients and volatile flavour compounds of fresh meat were determined at the College of Animal Science and Technology of Gansu Agricultural University and Qingdao Kechuang Quality Testing Co., Ltd., respectively. All assays were repeated in triplicate.

### 2.3 Determination of meat quality

#### 2.3.1 Determination of basic nutritional components

The crude protein content was determined using the semi‐micro Kjeldahl method (GB 5009.5‐2010). The fat content was determined using the Soxhlet extraction method (GB/T 5009.6‐2003). Protocols for the determination of protein (GB 5009.5‐2010) and fat levels in food (GB/T 5009.6‐2003) were issued by the General Administration of Quality Supervision, Inspection and Quarantine of the People's Republic of China (AQSIQ) and Standardization Administration of the People's Republic of China (SAC) in 2016.

#### 2.3.2 Determination of amino acid content and amino acid score

The amino acid content was determined using the determination method of amino acids in food (GB/T 5009.124‐2003). The tryptophan content was determined using the colorimetric method after alkaline hydrolysis of each sample. All measurements were repeated in triplicate.

Amino acid score (AAS) = the content of amino acid per gram nitrogen (or protein) of the tested protein/the content of amino acid per gram nitrogen (or protein) in the ideal model × 100.

#### 2.3.3 Determination of fatty acids content

The meat samples were pretreated according to the GB/T 17376‐2008. The fatty acid content was determined according to Wang et al. (2016).

### 2.4 Determination of flavour substances

#### 2.4.1 Determination of nucleotide and inosine monophosphate content

Nucleotide determination was performed according to the national food safety standard (the determination of nucleotides in infant food and dairy products). The content of inosine monophosphate (IMP) in different parts of fattening pigs was determined by high‐performance liquid chromatography (Wu et al., 2005).

#### 2.4.2 Determination of flavour substances

The volatile flavour substances in the meat samples were analysed by Qingdao Kechuang Quality Testing Co., Ltd. The volatile substances in fresh meat samples were pretreated with solid‐phase microextraction (SPME), and subsequently separated and identified by gas chromatography‐mass spectrometry (GC‐MS).

Sample processing method: First, 5 ml of meat sample was added to a 20 ml headspace bottle (to avoid exceeding 1/4 of the headspace bottle), and a tightened bottle cap was placed in the bottle. The headspace bottle was subsequently placed in the headspace at 55°C for 15 min. Lastly, the sample was extracted for 20 min by using the SPME needle with manual injection, and the extraction needle remained for 5 min at the injection port (Liu et al., 2017; SPME needle was aged at 250°C for 10 min before use. After cooling to room temperature, the SPME needle was washed with methanol, ethanol, ether, n‐hexane, deionized water, and methanol in turn).

Gas phase conditions: The TG‐5MS capillary column (30 m × 0.25 mm × 0.25 µm) was used as the chromatographic column. Helium gas was used as the carrier, and the flow rate was 1 ml/min, with no shunt. The injection temperature was 240°C and the column box temperature was 50°C. Temperature rising procedure: The initial temperature was maintained at 50°C for 2 min, and the temperature was increased to 240°C for 5 min at a heating rate of 4°C/min (Chen, 2004).

Mass spectrometry conditions: EI ionization, and the energy of ionization were 70 eV. The temperature of ionization and interface was 250°C. The mass scanning range was 40–450 m/z with an interval of 0.3 s (Wang et al., 2008).

### 2.5 Statistical analysis

Excel 2010 was used for data collection. The data are represented as mean ± standard deviation. Data were analysed using one‐way analysis of variance and Duncan testing using the SPSS 17.0 software, with *p* values < 0.05 considered to be statistically significant. All assays were repeated in triplicate.

## 3 RESULTS

### 3.1 Determination of meat quality

#### 3.1.1 Basic nutritional components

According to Table 2, all indicators were within the normal range. The content of crude protein, calcium, and phosphorus in tendon meat was higher than in *longissimus dorsi*, among which the phosphorus content was significantly different (*p* < 0.05). The intramuscular fat content of the rib muscle was higher than that in the *longissimus dorsi* and tendon meat (*p* < 0.05).

**TABLE 2:** Comparison of basic nutritional components in different parts of Du‐Ba binary hybrid pig muscle (g/100 g fresh meat sample)

Basic nutritional components	*Longissimus dorsi*	Rib muscle	Tendon meat
Crude protein	22.27 ± 2.31	22.50 ± 2.25	22.83 ± 2.12
Intramuscular fat	1.40 ± 0.11^b^	2.60 ± 0.18^a^	1.00 ± 0.08^b^
Moisture content	66.20 ± 6.89	64.83 ± 6.54	63.12 ± 7.12
Calcium	0.06 ± 0.01	0.07 ± 0.02	0.08 ± 0.01
Phosphorus	0.25 ± 0.01^b^	0.26 ± 0.01^b^	0.31 ± 0.01^a^

Note

“^a,b^”indicate the significant difference (*p* < 0.05).

#### 3.1.2 Amino acid content

The amino acid contents of different parts of the Du‐Ba binary hybrid pigs are presented in Table 3. A total of 17 amino acids were quantified in *longissimus dorsi*, rib muscle and tendon meat of Du‐Ba binary hybrid pigs, and no tryptophan was detected in any samples. The amino acid content of tendon meat was the highest. The proportion of essential amino acids and flavour‐associated amino acids in total amino acids per kilogram of *longissimus dorsi* were higher than those of the other two cuts. The proportion of glutamate in the total amino acids per kilogram of rib muscle was higher than that in the other two parts.

**TABLE 3:** The amino acid content in different parts of Du‐Ba binary hybrid pig muscle (g/kg)

Amino acid (AA)	*Longissimus dorsi*	Rib muscle	Tendon meat
Non‐essential amino acids			
^1^Asp	16.95 ± 1.56^a^	7.25 ± 0.75^b^	5.10 ± 0.45^c^
Ser	6.45 ± 0.57^a^	5.95 ± 071^b^	5.50 ± 0.46^bc^
^1^Glu	20.64 ± 1.85^a^	21.48 ± 1.97^a^	17.87 ± 1.62^b^
^1^Ala	9.54 ± 1.02	10.08 ± 1.11	10.02 ± 1.20
Semi‐essential amino acids			
Cys	4.42 ± 0.39b^a^	2.24 ± 0.15^b^	2.45 ± 0.32^b^
Arg	13.75 ± 1.31	12.33 ± 1.21	13.17 ± 1.32
Tyr	5.94 ± 5.24	5.94 ± 5.21	5.92 ± 5.62
^1^Gly	7.48 ± 0.65	7.74 ± 0.71	8.44 ± 0.72
^1^Pro	18.69 ± 1.89^c^	42.56 ± 4.20^b^	53.99 ± 5.12^a^
Essential amino acid (EAA)			
His	12.20 ± 1.15^a^	4.69 ± 0.36^b^	4.40 ± 0.41^b^
Lys	16.72 ± 1.59^a^	14.88 ± 1.12^b^	14.57 ± 1.23^b^
Met	4.83 ± 0.41	4.51 ± 0.38	4.77 ± 0.42
Thr	8.31 ± 0.78	8.56 ± 0.76	8.00 ± 0.82
Val	9.73 ± 0.92	10.30 ± 0.95	10.19 ± 1.20
Ile	8.89 ± 0.85	9.12 ± 0.88	8.84 ± 0.79
Leu	14.02 ± 1.32	14.57 ± 1.42	14.32 ± 1.32
Phe	7.16 ± 0.63	7.20 ± 0.71	7.23 ± 0.65
Total AA	185.72 ± 20.14	189.40 ± 20.31	194.78 ± 21.02
Total EAA	81.86 ± 6.75	73.83 ± 6.04	72.32 ± 6.14
Total flavour AA	58.82 ± 4.53^a^	48.80 ± 2.34^b^	44.58 ± 1.12^c^
EAA/TAA	44.08 ± 3.35	38.98 ± 2.97	37.13 ± 2.92
Glu/total AA (%)	11.11 ± 0.92	11.34 ± 0.97	9.17 ± 0.77
Flavor AA/total AA (%)	31.67 ± 2.25	25.77 ± 1.11	22.89 ± 0.53

Note

“^1^” was flavour amino acid; “^a,b,c^” indicate a significant difference (*p* < 0.05).

In the four non‐essential amino acids, the differences in the alanine content of the three parts were not significant (*p* > 0.05), and the content of aspartic acid and serine in the *longissimus dorsi* were higher than in the other two cuts. The cystine content in the *longissimus dorsi* was significantly higher than in the rib muscle and tendon meat (*p* < 0.05). According to the FAO/World Health Organization (WHO) model standard, EAA/TAA (essential amino acids/total amino acids) should be approximately 40% in the better protein composition. The EAA/TAA in the *longissimus dorsi* of Du‐Ba binary hybrid pigs was 44.08%, which is an ideal protein model.

As shown in Table 4, compared with the content of the essential amino acids in ideal protein proposed by FAO/WHO, the essential amino acids in *longissimus dorsi*, rib muscle, and tendon meat were lower than those in the ideal model, while the threonine and lysine contents approached the ideal model. The lysine score of the *longissimus dorsi* approached the ideal model.

**TABLE 4:** Essential amino acid scores in different parts of Du‐Ba binary hybrid pig muscle (mg/g pro)

Essential amino acid (EAA)	*Longissimus dorsi*	Rib muscle	Tendon meat	WHO recommendation mode (%)	Amino acid score of *longissimus dorsi* (%)	Amino acid score of rib muscle (%)	Amino acid score of tendon meat (%)
Met	4.83 ± 0.41	4.51 ± 0.38	4.77 ± 0.42	35.00	13.80 ± 1.17	12.89 ± 1.09	13.63 ± 1.20
Thr	8.31 ± 0.78	8.56 ± 0.76	8.00 ± 0.82	40.00	20.78 ± 1.95	21.40 ± 1.90	20.00 ± 2.05
Val	9.73 ± 0.92	10.30 ± 0.95	10.19 ± 1.20	50.00	19.46 ± 1.81	20.60 ± 1.90	20.38 ± 2.40
Ile	8.89 ± 0.85	9.12 ± 0.88	8.84 ± 0.79	40.00	22.23 ± 2.13	22.80 ± 2.20	22.10 ± 1.98
Leu	14.02 ± 1.32	14.57 ± 1.42	14.32 ± 1.32	70.00	20.03 ± 1.89	20.81 ± 2.03	20.46 ± 1.82
Phe	7.16 ± 0.63	7.20 ± 0.71	7.23 ± 0.65	60.00	11.93 ± 1.05	12.00 ± 1.18	12.05 ± 1.08
Lys	16.72 ± 1.59^a^	14.88 ± 1.12^b^	14.57 ± 1.23^b^	55.00	30.40 ± 2.89	27.05 ± 2.04	26.49 ± 2.24

Note

“^a,b^”indicate the significant difference (*p* < 0.05).

#### 3.1.3 Fatty acid composition

As illustrated in Table 5, the *longissimus dorsi* and rib muscles of the Du‐Ba binary hybrid pig included 15 saturated fatty acids and six unsaturated fatty acids. The tendon meat included 16 saturated fatty acids and seven unsaturated fatty acids. There were differences in the content of saturated and unsaturated fatty acids in the three parts (*p* < 0.05). The cholesterol content of the *longissimus dorsi* muscle was significantly higher than that of the rib muscle (*p* < 0.05). There was no significant difference between the cholesterol content of the *longissimus dorsi* and tendon meat (*p* > 0.05).

**TABLE 5:** Fatty acid composition in different parts of Du‐Ba binary hybrid pig muscle (mg/kg)

Fatty acid	*Longissimus dorsi*	Rib muscle	Tendon meat
Saturated fatty acids (SAFAs)			
C8:0	37.58 ± 3.15^a^	24.78 ± 2.26^b^	14.93 ± 1.58^c^
C10:0	9.11 ± 0.75^c^	34.07 ± 3.06^b^	64.70 ± 4.29^a^
C12:0	9.55 ± 0.67^c^	25.50 ± 2.34^b^	44.78 ± 4.12^a^
C13:0	6.44 ± 0.53^a^	3.65 ± 0.32^b^	1.87 ± 0.12^c^
C14:0	131.81 ± 10.24^c^	467.56 ± 24.28^b^	943.94 ± 46.65^a^
C14:1	3.84 ± 2.64^bc^	4.80 ± 3.36^b^	16.16 ± 1.25^a^
C15:0	9.75 ± 0.58^c^	15.19 ± 1.27^a^	14.58 ± 1.36^ab^
C15:1	100.91 ± 8.56^a^	99.35 ± 8.16^a^	66.72 ± 6.02^b^
C16:0	3,036.00 ± 102.34^c^	8,814.99 ± 154.28^b^	14,998.60 ± 198.38^a^
C16:1	492.65 ± 24.21^c^	1,621.65 ± 66.35^b^	3,786.70 ± 98.62^a^
C17:0	26.90 ± 1.28^c^	60.60 ± 4.35^b^	68.37 ± 5.82^a^
C17:1	15.32 ± 1.06^c^	48.02 ± 3.86^b^	88.71 ± 6.26^a^
C18:0	1,451.24 ± 56.86^c^	3,507.20 ± 75.64^b^	5,530.59 ± 95.56^a^
C20:0	5.71 ± 0.68^c^	25.80 ± 1.85^b^	63.01 ± 5.06^a^
C20:1	Not detected (<0.50)	50.47 ± 4.21^c^	174.63 ± 13.69^a^
C22:2	2.26 ± 1.02^a^	2.43 ± 1.26^a^	0.80 ± 0.06^b^
Unsaturated fatty acids (UFAs)			
C18:1N9C	1,148.90 ± 45.24^c^	2,063.42 ± 67.58^b^	3,907.99 ± 87.65^a^
C18:1N9T	Not detected (<0.50)	Not detected (<0.50)	Not detected (<0.50)
C18:2N6C	1,516.61 ± 51.26^c^	3,223.04 ± 80.28^b^	3,924.02 ± 88.57^a^
C18:2N6T	Not detected (<0.50)	Not detected (<0.50)	Not detected (<0.50)
C18:3N3	264.69 ± 13.54^c^	818.15 ± 24.32^b^	1,704.08 ± 48.95^a^
C18:3N6	9.88 ± 0.74^a^	6.86 ± 0.52^bc^	7.49 ± 0.56^b^
C20:3N3	Not detected (<0.50)	Not detected (<0.50)	Not detected (<0.50)
C20:3N6	Not detected (<0.50)	Not detected (<0.50)	48.49 ± 3.71
C20:4N6	379.37 ± 21.61^b^	444.55 ± 20.37^a^	342.42 ± 24.01^bc^
C20:5N3	Not detected (<0.50)	Not detected (<0.50)	Not detected (<0.50)
C22:1N9	Not detected (<0.50)	Not detected (<0.50)	Not detected (<0.50)
C22:6N3	Not detected (<0.50)	Not detected (<0.50)	v (<0.50)
Cholesterol	416.1181 ± 16.35^ab^	372.4187 ± 14.29^c^	428.9045 ± 18.92^a^

Note

“^a,b,c^”indicate a significant difference (*p* < 0.05).

### 3.2 Determination of flavour substances

#### 3.2.1 Nucleotide and inosine monophosphate

Table 6 shows that cytidine monophosphate (CMP) was not detected in the Du‐Ba binary hybrid pig, and the content of uridine monophosphate (UMP), guanine monophosphate (GMP) and IMP in the *longissimus dorsi* were the highest, in which the contents of UMP and IMP were significantly higher than in the rib muscle and tendon meat (*p* < 0.05). The adenosine monophosphate (AMP) content of the *longissimus dorsi* was significantly lower than those of the rib muscle and tendon meat (*p* < 0.05).

**TABLE 6:** Comparison of inosinic acid and related substances in different parts of Du‐Ba binary hybrid pig muscle (mg/100 g)

	*Longissimus dorsi*	Rib muscle	Tendon meat
CMP	Not detected (<0.17)	Not detected (<0.50)	Not detected (<0.17)
UMP	2.77 ± 0.12^a^	2.28 ± 0.09^b^	2.03 ± 0.15^bc^
GMP	1.47 ± 0.10	1.26 ± 0.08	1.29 ± 0.14
AMP	0.17 ± 0.01^b^	0.51 ± 0.02^a^	0.57 ± 0.01^a^
IMP	80.92 ± 6.36^a^	67.97 ± 5.03^b^	70.48 ± 5.45^b^

Note

“^a,b,c^”indicate a significant difference (*p* < 0.05).

#### 3.2.2 Flavour substances

As shown in Tables 7 and 8, there were differences between the retention times of volatile flavour compounds in the three parts of the Du‐Ba binary hybrid pig. A total of 43, 47 and 46 kinds of volatile flavour compounds were detected in the *longissimus dorsi*, rib muscle and tendon meat. Except for alcohols and aldehydes, the total content of volatile flavour compounds in the *longissimus dorsi* was the highest.

**TABLE 7:** Comparison of alcohols and aldehydes in different parts of Du‐Ba binary hybrid pig muscle

*Longissimus dorsi*		Rib muscle		Tendon meat	
Name	Relative content	Name	Relative content	Name	Relative content
Alcohols					
1‐Octen‐3‐ol	1.801 ± 0.272	Cyclobutanol	6.100 ± 0.747	1‐Octen‐3‐ol	2.062 ± 0.124
benzylalcohol	2.721 ± 0.425	Furfuryl alcohol	0.417 ± 0.023	benzylalcohol	2.337 ± 0.157
octanol	2.140 ± 0.371	n‐heptanol	1.398 ± 0.103	octanol	3.091 ± 0.205
2‐phenyl‐2‐propanol	0.444 ± 0.036	1‐Octen‐3‐ol	2.699 ± 0.267	2‐phenyl‐2‐butanol	0.321 ± 0.026
		benzylalcohol	2.165 ± 0.374	4‐Ethylcyclohexanol	0.287 ± 0.015
		octanol	3.192 ± 0.214		
		2‐phenyl‐2‐propanol	0.501 ± 0.013		
		1‐Nonanol	0.249 ± 0.008		
Aldehydes					
caproaldehyde	3.646 ± 0.363	pentanal	1.541 ± 0.092	3‐methyl butanal	0.698 ± 0.189
furfural	0.438 ± 0.026	caproaldehyde	13.087 ± 1.874	pentanal	1.280 ± 0.043
heptaldehyde	2.038 ± 0.097	furfural	0.231 ± 0.012	caproaldehyde	13.965 ± 0.846
benzaldehyde	4.002 ± 0.763	heptaldehyde	5.395 ± 0.380	furfural	0.334 ± 0.012
n‐capryl (ic) aldehyde	4.204 ± 0.356	trans‐2‐heptenal	0.913 ± 0.036	heptaldehyde	6.983 ± 0.573
phenylacetaldehyde	0.656 ± 0.075	benzaldehyde	4.412 ± 0.583	trans‐2‐heptenal	1.584 ± 0.024
trans‐oct‐2‐enal	1.587 ± 0.093	n‐capryl (ic) aldehyde	9.313 ± 0.692	benzaldehyde	2.955 ± 0.045
nonanal	9.862 ± 0.857	phenylacetaldehyde	0.464 ± 0.014	n‐capryl (ic) aldehyde	10.896 ± 0.952
trans‐2‐nonenal	0.828 ± 0.048	trans‐2‐decenal	1.985 ± 0.083	phenylacetaldehyde	0.594 ± 0.036
trans‐2‐decenal	1.488 ± 0.173	nonanal	15.594 ± 1.095	trans‐oct‐2‐enal	3.379 ± 0.267
trans‐2‐tridecenal	0.658 ± 0.068	trans‐2‐nonenal	1.040 ± 0.045	nonanal	14.979 ± 1.021
pyridine‐4‐carboxaldehyde	1.072 ± 0.075	cis‐4‐decenal	0.587 ± 0.086	trans‐2‐nonenal	2.539 ± 0.186
		Nonanal	0.596 ± 0.069	nonanal	0.406 ± 0.031
		trans‐2‐decenal	1.990 ± 0.328	trans,trans‐2,4‐nonadienal	0.220 ± 0.016
		trans,trans‐2,4‐decadienal	0.351 ± 0.015	trans‐2‐decenal	4.817 ± 0.236
		2‐undecenal	1.147 ± 0.282	dodecyl aldehyde	0.291 ± 0.006
		FEMA 2763	0.836 ± 0.013	trans,trans‐2,4‐decadienal	0.372 ± 0.054
		trans,trans‐2,4‐decadienal	0.363 ± 0.004	2‐undecenal	2.845 ± 0.189
		2‐undecenal	2.780 ± 0.157	FEMA 2763	0.442 ± 0.028
		FEMA 2763	0.432 ± 0.042		

**TABLE 8:** Comparison of volatile compounds in different parts of Du‐Ba binary hybrid pig muscle

*Longissimus dorsi*		Rib muscle		Tendon meat	
Name	Relative content	Name	Relative content	Name	Relative content
Carbondioxide	2.041 ± 0.124	2‐Nonanone	0.613 ± 0.036	Ethylene oxide	6.484 ± 0.297
Manganese [II] acetate, tetrahydrate	0.635 ± 0.014	oxaluricacid	0.393 ± 0.017	methylbenzene	0.516 ± 0.122
12‐(methylamino) dodecanoic acid	5.509 ± 0.263	Spiro[2.4]hepta‐4,6‐diene ≥95.0%	0.378 ± 0.027	Formic acid hydrazide	0.191 ± 0.021
2,3‐pentanedione	1.198 ± 0.065	o‐Xylene	0.989 ± 0.054	ethylbenzene	0.214 ± 0.018
di‐tert‐butylperoxide	0.365 ± 0.021	Styrene	2.164 ± 0.136	m‐Xylene	1.296 ± 0.034
methylbenzene	0.748 ± 0.039	2‐Oxime‐2‐methoxy‐phenyl	0.280 ± 0.009	Styrene	2.847 ± 0.342
o‐Xylene	0.497 ± 0.024	2,3‐Octadione	0.294 ± 0.033	Hexyl formate	1.832 ± 0.095
1,4‐dimethyl‐benzene	1.406 ± 0.076	3‐Octanone	0.252 ± 0.025	2,3‐Octadione	0.352 ± 0.048
2,2,4,4‐tetramethylpentanoic acid	0.788 ± 0.032	2‐pentylfuran	9.417 ± 0.863	2‐pentylfuran	4.912 ± 0.517
Styrene	4.165 ± 0.687	5‐Methyl‐3‐hexen‐2‐one	0.262 ± 0.015	(+)‐Cinene	0.533 ± 0.066
(Z)‐2‐Heptenal	0.763 ± 0.028	1‐methyl‐3‐propan‐2‐ylbenzene	0.170 ± 0.008	2‐Methyl‐2‐imidazoline	0.260 ± 0.041
TRANS‐4‐NONENE	0.495 ± 0.027	(+)‐Cinene	2.534 ± 0.347	1‐methyl‐1‐ethylcyclopentene	0.192 ± 0.011
2‐pentylfuran	3.949 ± 0.358	1‐methyl‐1‐ethylcyclopentene	0.184 ± 0.031	Caprylic acid	0.228 ± 0.017
7H‐Dibenzo[b,g]carbazole	0.557 ± 0.035	naphthalene	0.177 ± 0.006	1‐(2‐butoxyethoxy)‐ Ethanol	0.246 ± 0.008
(+)‐Cinene	1.321 ± 0.247	Bihexyl	0.342 ± 0.024	2‐n‐Heptylfuran	0.433 ± 0.025
Caprylic acid	0.446 ± 0.064	3‐Ethyl‐1,4‐hexadiene,	0.500 ± 0.067	1‐Hexadecene	0.348 ± 0.035
2‐Chloro‐4‐(4‐methoxyphenyl)pyrimidine	0.945 ± 0.102	tridecane	0.274 ± 0.015	2‐methyl‐5‐(1‐methylethenyl)‐Cyclohexanone	0.174 ± 0.009
Dimethylcarbamyl chloride	4.965 ± 0.325	1,1‐Dihydroxydodecane, ethylene glycol diacetate	0.420 ± 0.052	2‐Methylcyclopentyl alcohol	0.153 ± 0.006
NULL	1.024 ± 0.095	Azepino [1,2‐a]22139;,21545;"241823,2‐d]22055;"21878‐11‐372300, 5,6,7,8,9,11‐hexahydro‐2‐(4‐nitrophenyl)	0.580 ± 0.027	Cyclodecanone	0.153 ± 0.007
Palmitic acid	0.764 ± 0.030			2‐n‐Octylfuran	0.307 ± 0.042
Trimethylsilyl trimethylsiloxyacetate	0.391 ± 0.028			3,5,24‐Trimethyl‐Tetracontane	0.269 ± 0.031
(1‐methyl group ‐4 (1H)‐pyridylene)‐ methyl acetate	2.753 ± 0.137				
4‐Amino‐7H‐pyrrolo[2,3‐d]pyrimidine	2.453 ± 0.543				
2‐ (Trimethylsilylmethanol) Mercapto acetic acid	0.407 ± 0.024				
5H, 13H‐dibenzo [c, h] dipyrrolo [3,2‐e:3', 2'‐j] [2,6] diazanaphthalene, 6,7,7a,8,14,15‐hexahydro‐7,15‐dimethyl	1.409 ± 0.063				
Ethyl 4‐hydroxyphenylacetate	22.062 ± 3.238				
P‐Hydroxybenzene propanoic acid	0.363 ± 0.044				
	27		21		19
	98.93		88.34		96.05

## 4 DISCUSSION

Pork is one of the most consumed types of red meat in many countries worldwide. This study aimed to explore the differences in meat quality and volatile flavour substances between different cuts of pork, the results of which could provide the basis of consumer decisions when purchasing pork.

Pork has many advantages as a food product; high levels of protein and fat can provide nutrition and energy for the human body (Valenzuela et al., 2019). Pork quality can be determined by analysis of the nutrient content of pork samples; for example, intramuscular fat content directly determines physical factors such as pork juiciness and meat tenderness (Huang et al., 2017). Protein is an important component of organism tissues and organs, and is the direct carrier of human and animal life activities which plays a vital role in human growth and development (Drommer et al., 2015). Ma et al. found that the protein content of the *longissimus dorsi* in Luchuan‐Duroc binary hybrid pigs was 23.12% (Ma et al., 2015), while the crude protein content in Du‐Ba binary hybrid pigs was 22.27%; these two breeds therefore have almost the same level of proteins. Amino acid composition is not only one of the main indicators of meat protein nutrition, but is also an important factor affecting meat quality (Erkkilä et al., 2008). One previous study showed that the proportion of glutamate in total amino acids was the highest in Duroc‐Luchuan binary hybrid pigs, and the proportion of cystine in total amino acids was the lowest (Ma et al., 2015), which is in accordance with the results of this study. In addition, the study reported by Xi et al. revealed that the total essential amino acid content in the *longissimus dorsi* was higher than in the rib muscle of Bamei pigs (Xi et al., 2019), which is consistent with the results of this study.

Fatty acids are important nutrients and limiting factors in animal metabolism, growth, development and reproduction (Leggio et al., 2012). Essential fatty acids, including linoleic acid and linolenic acid, cannot be synthesized in the human body and must be obtained from food (Zhuang et al., 2018). Zhang et al. found that the protein and intramuscular fat content of Debao black pork were 22.20 g/100 g and 1.88%, respectively (Zhang et al., 2018), and the results of this study for the protein and intramuscular fat content of Du‐Ba binary hybrid pigs were similar to those of Zhang et al. (2018). In addition, the proportion of saturated fatty acids in pork was lower than in ruminants, while levels of polyunsaturated fatty acids were higher (Zhao et al., 2018). Cai et al. showed that the major fatty acids of the Hezuo pig were C16 and C18 fatty acids (Cai et al., 2004), which was consistent with the results of this study for the Du‐Ba binary hybrid pig. In addition, the intramuscular fat content of the rib muscle is higher than that of the *longissimus dorsi* and tendon meat. Xu et al. showed that the muscles around the shoulder of Duroc gilts have lower drip loss, muscle fibre diameter, shearing force, and higher intramuscular fat when compared with other cuts (Xu et al., 2004). However, a review of the literature has demonstrated that there are few studies on the differences in meat quality between different parts of pork. Thus, this study illustrates that the rib muscle has high levels of fat deposits, and tendon meat has a relatively high nutritional value.

Meat flavour is generally described as ‘umami;’ this flavour comes from the meat components, including free amino acids, small peptides, IMP and sugar (Yang et al., 2010). After livestock slaughtering, adenosine triphosphate (ATP) in muscle degrades continuously, and IMP is produced. IMP and other decomposition products in meat accumulated continuously, which made the meat taste thicker (Kitada et al., 1983). Xi et al. illustrated that the IMP content in the *longissimus dorsi* was higher than that in the rib muscle of the Bamei pig (Xi et al., 2019), and the IMP content was also higher than that in the rib muscle of the Du‐Ba binary hybrid pig. Hydrocarbons mainly refer to alkanes and olefins, which can be oxidized and gradually decomposed to produce aldehydes, ketones, and a small amount of esters; these improve the overall flavour of meat products (Al‐Thaiban et al., 2018). The difference in the relative content of aldehydes, alcohols and ketones is the most important reason for the difference in pork flavour (Pan et al., 2012). One previous study showed that the content of volatile flavour compounds (including aldehydes, alcohols, ketones) in the *longissimus dorsi* was higher than that of the rib muscle and tendon meat in PIC swine (Guo, 2009), which was consistent with the results of the Du‐Ba binary hybrid pig. Among the aldehydes, 2,4‐decadienal, which has a fat aroma, comprises a unique component of pork aroma (Sucan et al., 2002). Among the alcohols, 1‐Octen‐3‐ol has the flavour of ripe mushrooms, which contributes greatly to the flavour of pork (Kim & Kim, 2005). Guo et al. revealed that the flavour amino acid content in *longissimus dorsi* is higher than in other parts of ZFY pigs (Guo et al., 2009), which is consistent with the results of this study. Therefore, the findings indicate that the *longissimus dorsi* has a stronger flavour.

## 5 CONCLUSION

In conclusion, the rib muscle contains the largest fat deposits, the tendon meat has a relatively high nutritional value, while the *longissimus dorsi* has a stronger flavour. This study will provide a reference for breeding high‐quality pork breeds and the basis for consumers to purchase pork to meet consumer demand. However, this study has several limitations. First, this study used only a small number of experimental subjects owing to the influence of feeding cost, market price and other factors, which may affect the reliability of the results, and more studies are needed to verify this. Second, the limitation of the experimental conditions, such as extraction temperature, might affect the experimental results. Third, it is not sufficient to study the meat quality and flavour substances in *longissimus dorsi*, rib muscle and tendon meat; it will also be necessary to research other parts of the Du‐Ba binary hybrid pig.

## CONFLICT OF INTEREST

The authors declare that they have no conflicts of interest.

## AUTHOR CONTRIBUTION

**Guoshun Chen:** Conceptualization; Funding acquisition. **Yu Cai:** Software; Writing‐original draft. **Yingyu Su:** Formal analysis; Writing‐review & editing. **Dong Wang:** Software; Writing‐review & editing. **Xiaolong Pan:** Data curation. **Xijun Zhi:** Data curation.

## Funding information

This work was supported by National Natural Science Foundation of China (grant number 31960665) and Lanzhou Science and Technology Plan Project (grant number 2019‐4‐52).

## Ethics statement

The animal procedures used in this study were reviewed and approved by the Gansu Agricultural University's Academic Committee and the National Natural Science Foundation of China according to guidelines established by the Biological Studies Animal Care and Use Committee of Gansu Province (Approval No. 31660670).

### Peer Review

The peer review history for this article is available at https://publons.com/publon/10.1002/vms3.409.
